# Experimental study on flow and turbulence characteristics of jet impinging on cylinder using three-dimensional Lagrangian particle tracking velocimetry

**DOI:** 10.1038/s41598-023-37970-9

**Published:** 2023-07-06

**Authors:** Mirae Kim, Daniel Schanz, Matteo Novara, Philipp Godbersen, Eunseop Yeom, Andreas Schröder

**Affiliations:** 1 Rolls-Royce University Technology Center, Pusan National University, Busan, Republic of Korea; 2 Institute of Aerodynamics and Flow Technology, German Aerospace Center (DLR), Göttingen, Germany; 3 School of Mechanical Engineering, Pusan National University, Busan, Republic of Korea; 4 Eco-Friendly Smart Ship Parts Technology Innovation Center, Pusan National University, Busan, Republic of Korea

**Keywords:** Engineering, Mechanical engineering

## Abstract

When a round jet impinges on a convex cylindrical surface, complex three-dimensional (3D) flow structures occur, accompanied by the Coanda effect. To characterize the flow and turbulence properties of the general system, ensemble averages of 3D Lagrangian particle tracking velocimetry measurements were taken. The radial bin-averaging method was used in post-processing the tracked particles and corresponding instantaneous velocity vectors to generate appropriate ensemble-averaged statistics. Two impinging angles were selected, and at a fixed Reynolds number, the ensemble-averaged volumetric velocity field and turbulent stress tensor components were measured. The flow and turbulence characteristics of the impinging jet on the cylinder were notably different based on the impinging angle, especially in the downstream region. Surprisingly, the attached wall jet with a half-elliptic shape was abruptly thickened in the wall-normal direction, similar to the axis switching phenomenon observed in elliptic jets in the case of oblique impingement. In the jet-impinging region, the flow spread in all directions with high mean vorticity values. With the development of a 3D curved wall jet, both the Coanda effect and centrifugal force played a significant role in the flow behavior. A notable feature of the self-preserving region was the similarity of mean velocity profiles with scaling by the maximum velocity and the jet half-width for both impinging angle cases. Local isotropy of turbulent normal stresses was observed in this region, supporting the existence of self-preservation in the 3D curved wall jet. The volumetric ensemble-averaged Reynolds stress tensor revealed strong inhomogeneous turbulence in the boundary layer region and the curvature effect on the Reynolds shear stress in the free shear layer.

## Introduction

Impinging jets are a popular topic in flow measurement because they can be applied to any type of body and are easily implemented. An impinging jet can be characterized by the local transfer of heat and mass; therefore, it is used in a variety of industrial fields and design techniques. The round (circular) jet, which is the most common impinging jet shape, has been used as a basic research subject ranging from the local impinging area to large-scale structures. Kim et al. investigated the local flow and heat transfer phenomena based on the impinging angle and distance of the round jet^1^. Shademan et al. studied the behavior of large-scale vortex structures in the free jet and wall jet regions and their interaction with the wall shear stress^2^. The vortex dynamics in the jet boundary layer region on the flat surface of the round jet impingement were studied, and it was confirmed that a tertiary vortex was developed because of the interaction of the primary and secondary vortices in the wall jet region^3^. In addition to circular jets, studies on jets with various geometries, such as square, chevron, sweeping, and swirling, have also been conducted. Violato et al. compared the behavior of vortices formed by circular and chevron jets impinging on a plate^4^. Huhn et al. investigated time-resolved 3D flow structures of a turbulent jet impinging on a flat plate and assessed the accuracy of related pressure fields determined by *FlowFit* data assimilation^5^. The flow characteristics of the plane impingement of the feedback, feedback-free-type sweeping jets^6^. Wannassi and Monnoyer investigated the thermal flow characteristics of a complex flow with a staggered combination of conventional straight and swirling jets^7^. These interesting studies found that the flow characteristics of the impinging jet vary depending on the geometry type, number of jets, and the manner in which the fluid exits. However, most previous studies have been conducted with jets impinging on a flat surface.

In practice, jets often impinge on a surface with a curvature; therefore, studies on curved surface impingement are also required. Alnak et al. used numerical and experimental approaches to investigate the effects of flow dynamics and heat transfer caused by a water jet flow impinging on a cylinder^8^. In our previous studies, various physical properties of a round jet impinging on a convex cylinder were examined. Two-dimensional (2D) particle image velocimetry (PIV) measurements were performed to investigate the flow and turbulence characteristics of the curved wall jet in the streamwise direction^9^, and stereoscopic PIV measurements were performed to survey the 2D and three-component flow characteristics in the spanwise direction^10^. A large eddy simulation was conducted to visualize the evolution of the transient 3D vortex structure on the cylinder^11^. However, these previous studies were based on 2D flow field measurements or computational fluid dynamics; in order to achieve a more complete picture of the related fluid dynamics and explain their findings it is necessary to obtain a detailed three-dimensional (3D) flow field in a well-controlled experiment.

Because 2D wall jet flows have characteristics of a wall boundary layer and free shear flow simultaneously, it has long been studied as a canonical flow of turbulence. The jets eject parallel to the plate from the 2D slot develop along the wall and have downstream self-preservation characteristics. Subsequently, the slot jet ejected in the tangential direction to the cylinder wall was named a 2D curved wall jet, and experimental studies were conducted^12,13^. The similarity of the average velocity profiles was clearly observed, revealing the existence of self-preserving characteristics. It was also found that the Coanda effect was large along the cylinder surface, delaying flow separation and causing the jet to stay attached to the wall.

When the round jet impinges on a circular cylinder wall, the flow develops in form of a wall jet with a characteristic 3D flow field along the curved surface of the cylinder. These types of flows frequently appear in engineering applications. Kim et al. named this flow a 3D curved wall jet and presented the time-averaged flow characteristics measured by a 2D PIV experiment^9^. It is a simple geometric structure in which a round jet impinges in the circumferential direction with a 2D cylinder wall; however, after the jet impingement, high momentum fluid flows not only in the circumferential direction but also in the axial direction of the cylinder, developing a complex 3D flow structure with corresponding pressure gradients. Additionally, the dynamic velocity range in this flow is very large. The results were represented by combining several fields of view for using the 2D PIV technique; however, it is difficult to determine whether the flow characteristics of the 3D curved wall jet were accurately identified.

A 3D curved wall jet can be included as a canonical form of wall turbulence. However, many questions remain. First, what are the kinematic characteristics when the round jet impinges on the circumferential wall of the cylinder and does it spread radially like a jet impinging on a flat plate? Second, because the wall jet flows along the convex surface, where is the location of the separation point and what is the effect of the impinging angle on the separation point? Third, is the 3D curved wall jet similar in mean velocity distribution, and do self-preserving properties exist? The only way to answer all these questions is to measure the entire 3D velocity field simultaneously.

Flow field measurements have progressed considerably owing to the development of measurement techniques, data processing algorithms, and their applications in various fields. The representative technique for flow field measurement is the PIV. It has been developed over almost 40 years^14^. The measurement domain was developed from local points to 2D and 3D flow fields. Until recently, volumetric illumination, increasing seeding density, and reconstruction technologies have been developed to enable the measurement of 3D instantaneous flow fields with sufficient resolution to analyze flow structures with multiple-scales in turbulence. Lagrangian particle tracking (LPT) is one of the most successful methods in recent years. The LPT method can track and predict the trajectories of time-resolved particles in a 3D flow field, and it is possible to measure small-scale turbulence structures^15^. Nowadays, iterative particle reconstruction and Shake-The-Box are mainly used as algorithms for LPT techniques^16–18^. IPR determines instantaneous 3D particle distributions from few camera projections by improving iteratively the accuracy of the actual particle locations and removing ghost particles within repeated triangulation processes. STB uses these particle positions over several time steps to obtain initial particle tracks which are used for a predictor–corrector scheme refining them by “shaking” the particle positions in space after each time-step by means of simultaneous re-projections onto the original images. The STB processing is progressively adding new particle tracks over time and is paving its way through long time series of particle images in an efficient manner after convergence of the 3D particle tracking system is reached. These highly advanced flow measurement technologies enable the measurement of complex 3D flow fields and the undertaking of various analyses^19^. In our previous study, time-resolved volumetric LPT measurements were conducted using a Universal Robots-UR5 robotic arm with four CMOS cameras to obtain the 3D flow structure on the cylinder^1^; however, it had the drawback of low particle density. Instead of the tracer particles exiting the jet, the entrained particles from the wind tunnel were used to measure the jet impingement on a cylinder, hindering the observation of the small-scale flow structure around the cylinder.

In this study, a 3D flow field was obtained with a single LPT measurement using four high-speed cameras in a water jet facility to further explore the hidden physics in the impinging jet flow around a cylinder. Additionally, to estimate the flow along the curved surface appropriately, a radial bin-averaging method has been proposed. Using the proposed binning method, various interpretations were accomplished on the flow and turbulence characteristics associated with a 3D curved wall jet on a circular cylinder, which was difficult to confirm with the conventional binning method. This study aims to analyze the fluid mechanical characteristics of the ensemble mean and variance of the impinging jet flow near a curved surface. The effects of the impinging angle on the 3D flow and turbulence characteristics were also examined.

## Materials and methods

### 3D LPT

**Figure 1:**
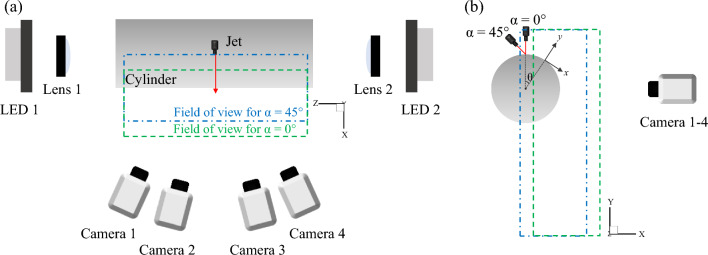
Experimental setup and coordinate systems for the time-resolved 3D LPT measurement: (**a**) top view and (**b**) side view.

Figure 1 shows the experimental setup for the time-resolved 3D LPT. A 3 mm-diameter (d) round jet nozzle and a 52 mm-diameter (D) circular cylinder were used for the flow measurement. The jet nozzle and cylinder were placed in a 16-faces water tank with tetradecagonal sides. 40 μm-diameter *Orgasol* polyamide powder was used as seeding particles. During the experiment, water containing seeding particles was supplied to the jet nozzle and water tank. The jet impinging angle (α) was 0° and 45°, and the height of the jet above the impingement point of the cylinder surface was fixed at 12 mm for both impinging angles. The Reynolds number (Re) was defined as (4 $\dot{\mathrm{V}}$/πd^2^)d/ν, where $\dot{\mathrm{V}}$ is the volumetric flow rate and ν is the kinematic viscosity, and Re was fixed at 17,875. The maximum jet exit velocity (U_jet_) was approximately 6 m/s. Four Phantom v2640 high-speed cameras with f = 60 mm Carl Zeiss Makro-Planar lenses in Scheimpflug mounts operated at 4 kHz were used to capture the particle images, which were cropped to a resolution of 1152 × 1952 pixels. The angle between cameras 1 and 4 was 89°. Two arrays of high-power white LEDs with individual collimating lenses were used for volumetric illumination. The light was further focused with two 1,000 mm focal length lenses placed on either side of the water tank and a passé-part-tout finally served as a rectangular knife-edge. The commonly illuminated and imaged measurement volume was approximately 80 × 140 × 50 mm^3^. The measurement volume consisted of half of the lateral cylinder and the wake area below the cylinder. To improve the experimental accessibility, Fig. 1b shows that the field of view was moved 10 mm in the X direction when the impingement angle was 0°. X, Y, and Z represent the frontal, vertical, and spanwise directions in the fixed coordinate system, respectively. Additionally, the curvilinear (moving) coordinate system according to the cylinder angle (θ) was applied, where *x*, *y*, and *z* represent the streamwise, wall-normal, and spanwise (transverse) directions, respectively.

In order to accurately triangulate the positions of particles from all 2D image projections in the 3D space, the imaging system was optically calibrated before capturing the particle images during the flow measurement. A two-plane calibration plate (*LaVision type 309–15*) was placed at the cylinder position for 3D image calibration. Geometric calibration was further refined using volume self-calibration and calibration of the optical transfer function (OTF)^16,20^. The initial error, in the order of 1 pixel, was reduced to approximately 0.1 pixels after volume self-calibration.

After particle image acquisition, the LPT process was performed based on the variable time-step STB (VT-STB) method^15^. In the flow of the jet impinging on the cylinder, the particle velocity at the jet outlet was approximately 6 m/s, and the particle velocity outside the wall jet was approximately 0.01 m/s. Additionally, the jet nozzle diameter was small compared to the cylinder diameter and full measurement volume, and thus the flow field had a very high dynamic velocity range. Therefore, the VT-STB method, which works with multiple time separations, was used to ensure optimal particle tracking in all velocity regions. VT-STB begins its evaluation with a time separation that can optimally track the slowest particles in the flow, and then tracks faster particles with each iteration as the time separation decreases iteratively. Finally, only the fastest remaining particles are tracked, reaching the original time separation of the imaging frame rate. The method showed distinct advantages over standard STB processing with the ability to track particles in different velocity regions with optimal time separation, in the reduction of ghost tracks, and the tracking accuracy.

A position spectrum analysis of the tracked particles was performed to obtain the position accuracy for the uncertainty analysis and for determining the cross-over frequency between signal and noise allowing for an optimal temporal TrackFit using 3rd order B-splines^21^: Using an initial run of VT-STB for α = 0°, the power spectrum of the raw tracks was determined independently for the X, Y, and Z components. The average position accuracy can then be read as the value of the plateau, which is reached after the measurement signal reaches the noise level^21^. The determined mean position accuracies at the X, Y, and Z positions were 10, 8, and 14 μm, respectively. After fitting, the accuracy usually improves by an approximate factor of 2, resulting in accuracies of 8, 4, and 7 μm, respectively.

**Figure 2:**
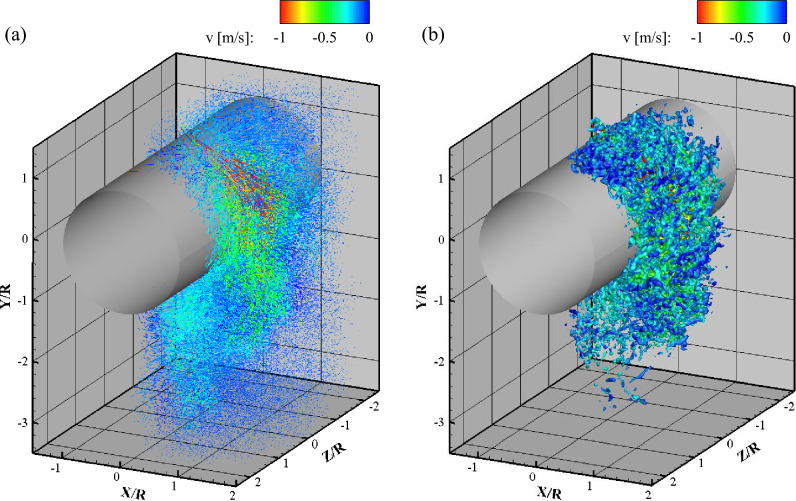
Full-scale instantaneous velocity and vorticity fields obtained by the 3D LPT by VT-STB: (**a**) Lagrangian particle tracks and (**b**) isosurfaces of Q-criterion (10,000 s^-2^) after FlowFit data assimilation colored by v-velocity.

Figure 2 shows the full-scale instantaneous 3D velocity and vorticity fields obtained by the VT-STB method and subsequent FlowFit data assimilation for α = 45° at steady state. The Lagrangian particle tracks (Fig. 2a) indicate that the impinged jet spreads transversely, while the high-speed wall jet moves in the streamwise direction attached to the curved cylinder surface. The spread-out jet reverted to the center of the streamwise axis before separation. Additionally, the Coanda effect on the 3D wall jet flow along the cylinder wall is vividly shown with a delay of separation up to an azimuthal angle of 180°. The downstream flow is highly turbulent with large-scale motions. Figure 2b shows the result of applying the FlowFit algorithm to the Lagrangian particle tracks^21^. FlowFit is a regularized interpolation onto the Eulerian regular grid, fitting the unstructured particle data to a structured grid of cubic B-splines with a 0.7 mm spacing, while applying physical regularizations. The vortex structures are represented by the iso-surfaces of the second invariant of the velocity gradient tensor, Q. The instantaneous flow field is very complex (Fig. 2). Retrieving meaningful flow physics from large-scale datasets of randomly distributed velocity vectors by LPT is a challenge. Following the traditional approach, this study firstly focuses on the hydrodynamic properties of the ensemble-averaged flow and turbulence fields based on large-scale and complex time-resolved 3D velocity field measurement data. For the full-scale instantaneous Lagrangian particle tracks in the transient case, see Movie 1 in Supplementary material.

### Radial bin-averaging process

**Figure 3:**
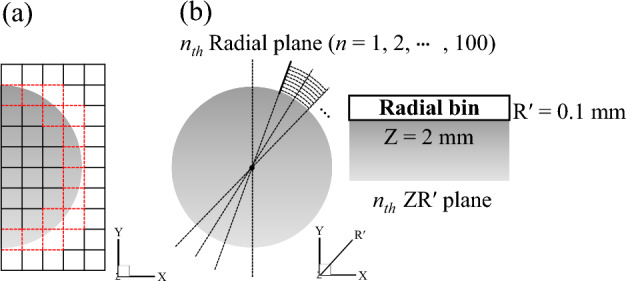
Configuration of bins around the cylinder surface: (**a**) Cartesian bin and (**b**) radial bin.

The bin-averaging method is a direct statistical approach that allows statistical convergence up to a certain threshold while maintaining the spatial resolution of the tracked particle data^15^. Generally, a Cartesian coordinate system is used in the bin-averaging step of data post-processing. However, the current experiment was performed on a cylinder with a circular curvature. Therefore, in this study, the radial bin-averaging method was applied for the appropriate conversion of scattered instantaneous velocities to a gridded mean value distribution and corresponding fluctuation velocity statistics. Additionally, it is worth analyzing the difference between the bin-averaging results of the Cartesian and radial coordinates for curved wall jets around the convex cylinder. Bin-averaging was performed for all 45,407 time steps to obtain ensemble-averaged data. The resolution of the Cartesian grid used for bin-averaging was 0.5^3^ mm^3^. The spatial resolution of the ensemble-averaged velocity field was not significantly affected when the linear bin size was less than half of the local radius of curvature^22^. The bin size (0.5 mm) used was considerably less than half of the local radius of curvature (13 mm), ensuring sufficient spatial resolution. However, there was still a mismatch between the fluid mesh and the cylinder surface, as indicated by the red dashed line in Fig. 3a. To completely eliminate these discrepancies, the radial grid bin-averaging method was proposed to create a radial bin around the cylinder, such as a mesh in computational fluid dynamics. Figure 3b shows the radial bin used in this study. The radial plane represents the plane of the component in the radial direction (R' $=\sqrt{{\mathrm{X}}^{2}+{\mathrm{Y}}^{2}})$ and the spanwise direction (Z) according to the cylinder angle. The radial bin has a height R' = 0.1 mm and width Z = 2 mm in the radial plane with the cylinder angle (2π) divided by 100. The number of elements (I, J, and K) indexed was 160, 280, and 100, respectively, for the Cartesian grid and 40, 650, and 62, respectively, for the radial grid. The selected radial bin was the optimal bin determined by comparing the results of various radial bins. The cylinder angle had the largest influence on the shape of the radial bin. After the cylinder angle was selected, the radial height and the spanwise width were determined by considering the calculation time and the physical properties to be analyzed.

## Results and discussion

### Comparison of the flow fields for Cartesian and radial bin-averaging methods

**Figure 4:**
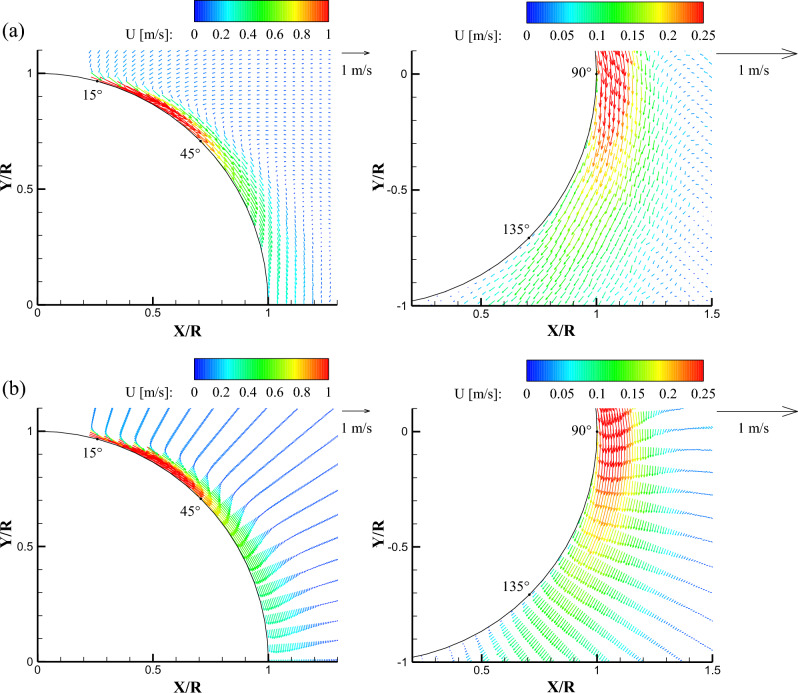
Mean velocity vector fields in the center plane (XY plane at Z = 0) obtained at α = 0° using (**a**) Cartesian binning and (**b**) radial binning.

**Figure 5:**
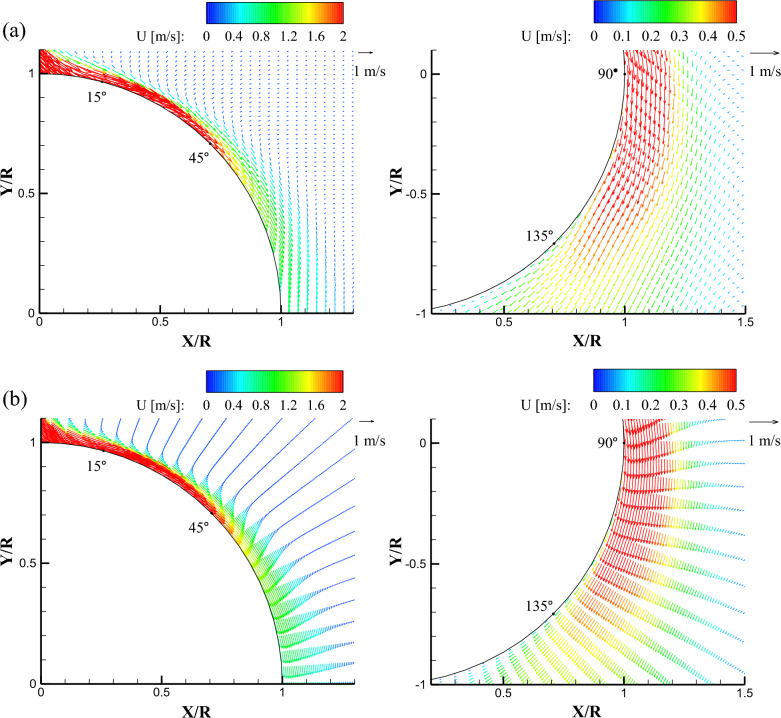
Mean velocity vector fields in the center plane (XY plane at Z = 0) obtained at α = 45° using (**a**) Cartesian binning and (**b**) radial binning.

Figures 4, 5 show the ensemble-averaged total mean velocity ($\mathrm{U}=\sqrt{{u}^{2}+{v}^{2}+{w}^{2}}$) vector fields at the center of a streamwise plane with impinging angles of 0° and 45°, respectively. To more clearly visualize the velocity vectors and identify the difference between the binning methods, the velocity vectors were skipped with intervals of 2 in the upper (0° < θ < 90°) area (left-hand side in Figs. 4 and 5) and 3 in the lower (80° < θ < 165°) area (right-hand side in Figs. 4 and 5). Note that the skip interval value of 2 represents the velocity vectors at every second data point, and 3 represents the velocity vectors at every third data point. Also, the color scale of the contour was different because the velocity difference between the upper and lower regions of the flow field is large. In all binning methods, the relatively fast curved wall jets adhered to the wall until θ was approximately 45°, in the case of a zero impinging angle, as shown in Fig. 4. The mean streamline of the curved wall jet began to move away from the cylinder wall at θ = 105°. In the Cartesian binning, when θ exceeded 60° as the curved wall jet slowed down, the vector did not follow the curve smoothly anymore. However, the entrainment of the flow around the curved wall jet was observed in the Cartesian binning. In contrast, in the radial binning, the velocity profile according to θ clearly shows the flow physics of the curved wall jet. Because the number of elements was smaller than that of the Cartesian grid, a void was observed in the interspace between the two radial lines, and entrainment of the flow around the curved wall jet was not resolved sufficiently.

When the jet impinges at 45°, a thicker curved wall jet develops compared to the normal impingement, as shown in Fig. 5. The high-momentum region near the surface was longer than that of the normal impingement for all binning methods. In the Cartesian grid, the velocity vectors were directed vertically in the region where the cylinder angle was from 90° to 110°, complicating the physical interpretation, as compared to that in the radial grid. The mean streamline moved away from the cylinder wall at approximately θ = 125°, and the curved wall jet attachment sustained approximately 20° longer than the normal impingement. The mean velocity vector field analysis demonstrated that the Cartesian grid was appropriate for the analysis of the entire measurement volume, and the radial grid was adequate for the intuitive visualization of the wall jet profiles and the flow analysis of small structures, such as the curved wall jet along the curvature.

**Figure 6:**
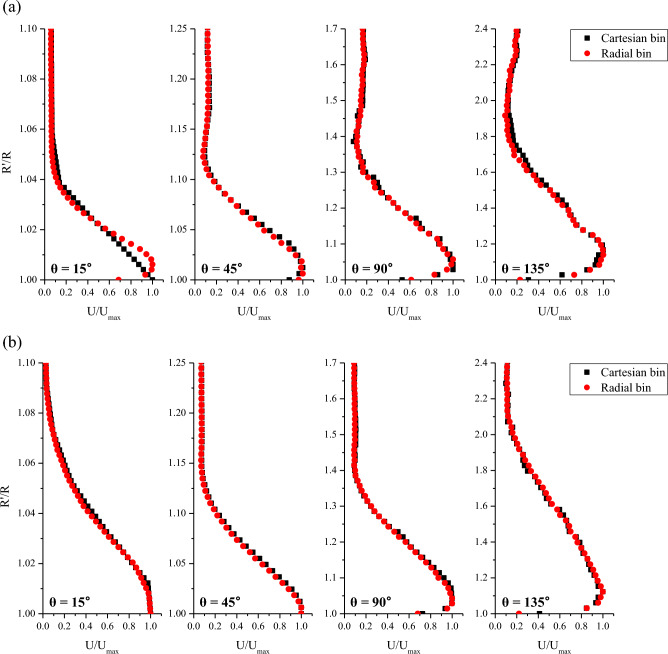
Mean velocity profiles along the wall-normal direction at various θ positions in the center plane for (**a**) α = 0° and (**b**) α = 45°. Red circle data were obtained by the radial binning while black rectangle data were extracted by the Cartesian binning.

Figure 6 shows the line data of the mean velocity profiles of the curved wall jet along the cylinder wall for the two impinging angles. The abscissa axis represents the mean velocity magnitude (U) normalized to the maximum mean velocity (U_max_), and the vertical axis represents the radial distance (R′) normalized by the cylinder radius (R). The radial distance from the wall to the position at which the maximum velocity appears represents the inner shear layer (boundary layer region), and the region outside the boundary layer represents the outer shear layer (wall jet region). In the 0° impingement angle measurement, the point where θ = 15° was located at the edge of the measurement area. Therefore, as indicated in Fig. 6a, at θ = 15° the velocity profiles show a strong dependency on the binning method. Cartesian binning provided incorrect information, but the radial grid showed a good velocity profile. When the grid is radially shaped, it is possible to represent fast particles (velocity vectors) along the radial direction (that is, curvature) better than in the case of a dense Cartesian grid. Downstream, the mean velocity profiles coincided well regardless of the binning method. At θ = 135°, the velocity profile became unstable, and the wall jet thickness abruptly increased to R′ = 1.8 when α = 0°. Figure 6b shows the line data of the mean velocity profiles at the center plane when the round jet impinges at 45°. When the impinging angle was 0°, the maximum velocity point appeared higher up the cylinder wall because the longitudinal momentum in the flow direction was rapidly reduced by the lateral momentum flux. The wall jet region was wider overall when the impingement angle was 45° than when the impingement angle was 0°, especially near the impinging area (θ = 15°) and the last azimuthal plane (θ = 135°). Additionally, because of the influence of the initial momentum of the jet flow and the intensity of turbulence around the cylinder, the mean velocity profiles according to the binning method were better fitted when α = 45°. It should be noted that the shear layer developed up to R′ = 2.1 when α = 45°. Table 1 presents the standard deviation (%) of the mean velocity between Cartesian and radial binning at the same R'/R point. The standard deviation values were 4% or less, except when α = 0° and θ = 15°, which was located at the field of view edge. Because the deviation is relatively small, the subsequent line data and figures are shown only for the radial binning. The purpose of this work is showing that the conventional Cartesian binning causes significant errors in strongly curved flows. The reason is that there is a severe velocity gradient in the Cartesian binning compared to that in the radial binning in the wall jet flow along the cylindrical surface. If we can find a general rule to calculate the uncertainty in the grid transformation, the rule could be useful for the post processing of Lagrangian particle tracking velocity vector measurement. However, the uncertainty is caused by not only geometrical property but also flow characteristics. It should be our further research topic.

**Table 1:** Standard deviation (%) of the mean velocity between Cartesian and radial binning.

	θ = 15°	θ = 45°	θ = 90°	θ = 135°
α = 0°	6.38	2.21	2.23	3.82
α = 45°	1.27	1.13	1.40	3.14

### Ensemble-averaged flow and turbulence characteristics for radial binning

#### Features in the center plane

**Figure 7:**
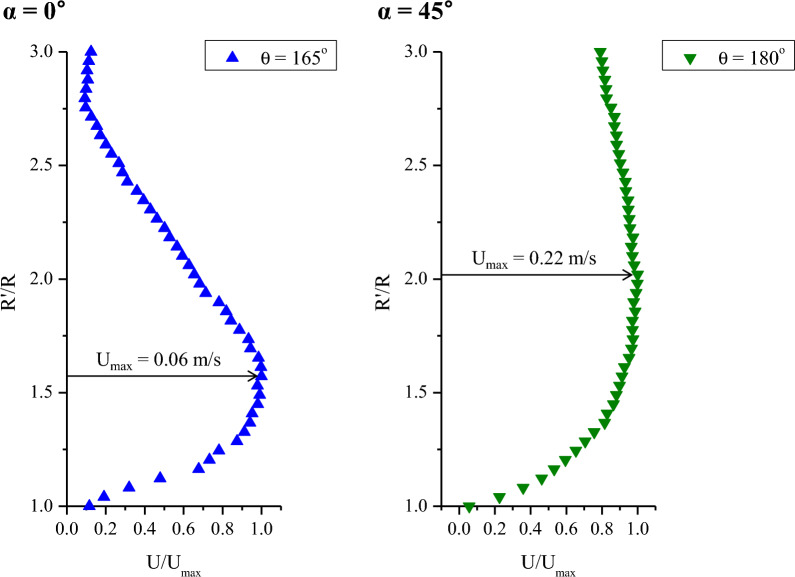
Mean velocity profiles at the maximum downstream point of the measurement center plane.

At the same Re, the flow produced on the cylinder surface differed significantly according to the impinging angle. In the case of normal impingement, the jet is divided in half to the front and back of the cylinder surface. However, at an impinging angle of 45°, the jet flows along the cylinder wall in the direction in which most of the fluid is moving. When a uniform flow passes a 2D cylinder, separation of the flow occurs at an azimuthal angle of 85° to 105° owing to the presence of a reverse pressure gradient depending on the Re. However, it was found that when the round jet flowed along the cylinder wall, the flow was attached to the back side of the cylinder for a long time owing to the Coanda effect (see Fig. 2). Figure 7 shows the mean velocity profiles at the maximum downstream point in each measurement center plane of the two impinging angles. The first y-directional bin size of radial binning was 0.1 mm so that the minimum distance from the wall was 0.05 mm (50 μm) for the velocity vector measurement. At both impinging angles, the wall jet shear layer was much thicker compared to Fig. 6. The delay of flow separation due to the Coanda effect can be clearly seen through the presence of the location of the maximum velocity inside the wall jet shear layer.

**Figure 8:**
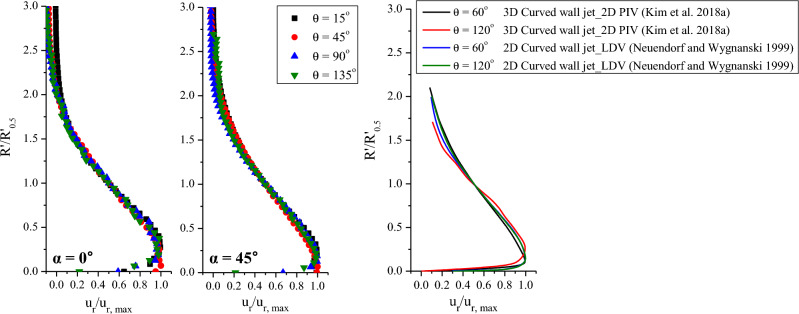
Similarity comparison of the non-dimensional mean streamwise velocity profiles.

The mean velocity profile of the wall jet shows a profile in which the boundary layer shapes from the wall to the maximum velocity point. Additionally, it is combined with the free-jet flow shape from the maximum velocity to the free-stream flow. It is well known that the time-averaged velocity distribution in the self-preserving region of a 2D plane wall jet or a curved wall jet coincides with a single curve when the maximum velocity and half width of the jet are scaled. However, the thickness of the 3D curved wall jet is three-dimensionally different, and the flow direction is 3D. Previous studies stated that the self-preserving phenomenon could not occur in a 3D curved wall jet^9^. Figure 8 shows the similarity of the 3D curved wall jet normalized to the maximum streamwise velocity (u_r, max_) and the jet half-width (R′_0.5_). The position of the jet half-width denotes the radial distance, where the mean streamwise velocity (u_r_) decreases to half of the maximum streamwise velocity value. Note that the streamwise velocity is the velocity component in the curvilinear coordinate system. Also, the similarity trend was compared with the mean velocity profile of the 3D curved wall jet measured by 2D PIV and the 2D curved wall jet measured by Laser-Doppler Anemometer^11,13^. In contrast to previous 2D PIV measurements, the similarity of the mean velocity profiles of the 3D curved wall jet at both impinging angles was well matched with the data of the 2D curved wall jet. Self-similarity refers to an asymptotic state in which the statistical properties are independent of the initial conditions and each variable and is achieved with a specific flow. The evidence of self-preservation is similarity profiles with appropriate scales^23^. It is very interesting to be able to discover the self-preserving phenomenon of 3D curved wall jets with advances in measurement and data processing technologies.

**Figure 9:**
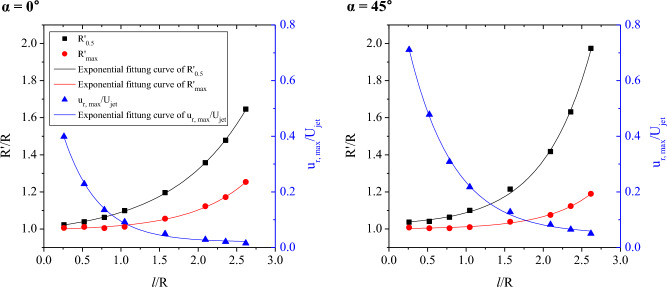
Variation of the jet half width (R′_0.5_), the position of the maximum streamwise velocity (R′_max_), and the normalized maximum streamwise velocity (u_r, max_/U_jet_) along the cylinder arc distance.

Figure 9 presents the development of the jet half width and the position of the maximum streamwise velocity (R′_max_) according to the impinging and cylinder angles. Additionally, the decay of the maximum streamwise velocity along the streamwise direction is shown for both impinging angles. The vertical axis on the left shows the position of the jet half width and maximum velocity, and the vertical axis on the right shows the normalized maximum streamwise velocity. The abscissa axis is represented by the cylinder arc distance (*l* = Rθ) normalized to the radius. As the impinging jet flowed over the surface of the cylinder, the jet half width widened. The difference in the jet half width with respect to the impinging angle was greatest in the downstream region, and the variation in the jet half width with respect to the cylinder angle was very clear when the impinging angle was larger. In contrast, the variation in the maximum streamwise velocity position was greater for normal impingement because the respective fraction of the momentum was smaller in the normal impingement. Therefore, the maximum streamwise velocity is further away from the cylinder wall, so the rate of change is greater, and the thickness of the curved wall jet is thinner, so the rate of change of the jet half width is small.

The growth rate of the wall jet depends on the entrainment rate, and the jet half-width of the 2D plane wall jet increases linearly^24^. However, in the case of a 2D curved wall jet, the growth rate is nonlinear and varies with the radius of curvature^13^. For a 3D curved wall jet, the growth rate of the half-width and entrainment is 3D. A theoretical approach to the entrainment mechanism should be examined in the future, as this topic is beyond the scope of this paper. Based on the experimental data, an empirical formula for the half-width growth and maximum velocity point along the centerline was obtained. As shown in Fig. 9, the exponential fitting curve is the best. The formula applied to the exponential fitting was:

1 $$y={y}_{0}+A{e}^{{R}_{0}x}$$

where *y* denotes R′_0.5_/R or R′_max_/R or u_r, max_/U_jet_ and *x* denotes *l*/R. Table 2 presents the correlation coefficient R^2^, which indicates the goodness of fit, and the slope of the fitting curve R_0_ for each impinging angle. R_0_ at the jet half width and the position of the maximum streamwise velocity, was 1.04 and 1.38 at α = 0°, respectively, and 1.61 and 1.67 at α = 45°, respectively; thus, the similarity is consistent, especially for oblique impingement. The decay of the maximum streamwise velocity also fits well with the exponential function. When a jet impinges with an angle at the same Re, the rate of decrease of the maximum streamwise velocity is greater because the faster wall jet flows along the curved surface.

**Table 2:** Correlation coefficient (R^2^) and slope of the exponential fitting curve (R_0_) for each impinging angle.

	α = 0°	α = 45°
R^2^ @R′_0.5_	99.98%	99.86%
R_0_ @R′_0.5_	1.04	1.61
R^2^ @R′_max_	99.51%	99.55%
R_0_ @R′_max_	1.38	1.67
R^2^ @u_r, max_/U_jet_	99.80%	99.87%
R_0_ @u_r, max_/U_jet_	− 2.19	− 1.72

**Figure 10:**
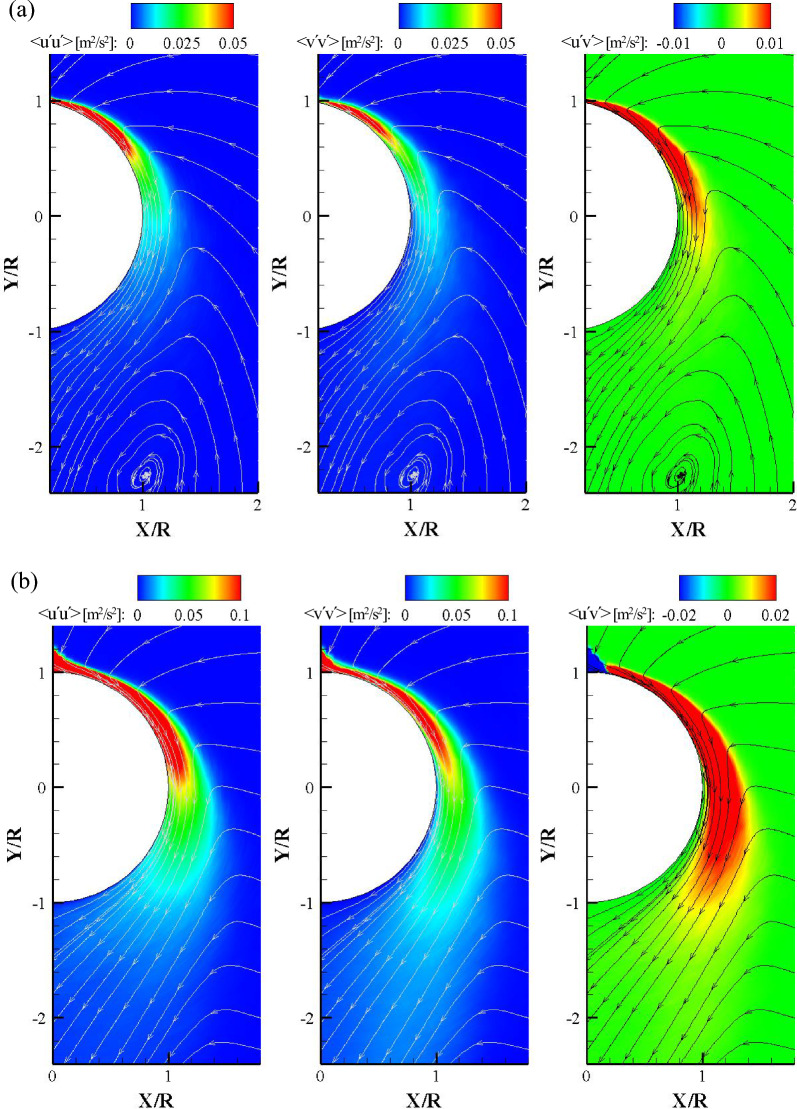
Contours of the Reynolds stress components with streamlines in the center plane for (**a**) α = 0° and (**b**) α = 45°.

Figure 10 shows the Reynolds stress contours with streamlines at the center plane. All turbulence fluctuation components, that is, u′, v′, w′, and the Reynolds stress tensor, were obtained from a curvilinear coordinate system and a radial grid. Note that the contour is denoted by the m^2^/s^2^ unit owing to the high dynamic velocity range and to directly compare the stress components and impinging angles. The development of turbulent stresses in the streamwise direction was significantly different with respect to the impinging angle. The Reynolds normal stresses < u′u′ > and < v′v′ > were five times higher than those of the Reynolds shear stress < u′v′ > . When α = 0°, the high-magnitude regions of turbulent stresses were distributed up to θ = 110°, and a swirl of the streamline was formed downstream of the cylinder. The streamlines toward the cylinder (wall jet region) are attributed to the entrainment process, whereas the large vortex downstream of the cylinder could be induced by the attached wall jet. The vortex centered at X/R = 1.0, Y/R =  −  2.3 formed with closed streamlines, so that the swirling motion could not contribute to fluid entrainment into the wall jet. For α = 45°, the high-magnitude regions of turbulent stresses were elongated further to θ = 170°. Normal stresses showed similar contours and magnitudes, but the streamwise normal stress < u′u′ > contour was thicker than that of < v′v′ > . This can be explained by the streamwise momentum being dominant in the wall jet. A negative < u′v′ > region was visible near the oblique impinging point. The wall jet flowed over the convex curved surface, but the jet at the impinging point had concave streamlines with a small radius of curvature. This change in curvature at the impinging point leads to a sign change in < u′v′ > . Unlike in the case of α = 0°, there was no isolated vortex motion and streamlines from the ambient moved to the wall jet, even far downstream of the cylinder. It is obvious that the surrounding flow in oblique impingement was more strongly entrained toward the curved wall jet.

**Figure 11:**
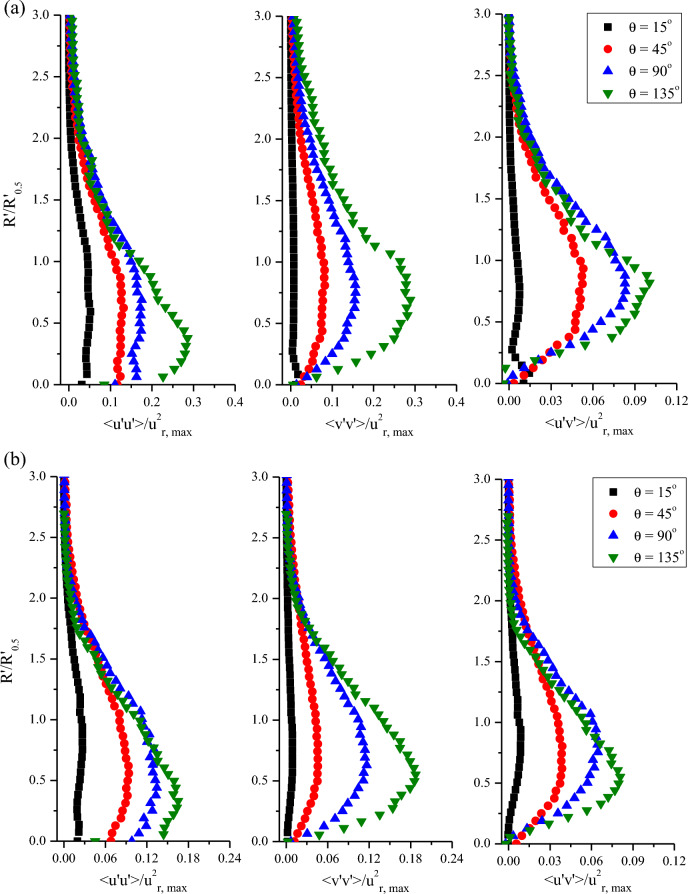
Normalized Reynolds stress profiles for (**a**) α = 0° and (**b**) α = 45°.

Figure 11 shows the normalized turbulent normal and shear stress profiles according to the impinging and the cylinder angles. The Reynolds stresses were normalized to the square of the maximum streamwise velocity at the azimuthal planes. Note that the reason for the higher dimensionless value of Reynolds stress in normal impingement is that the maximum streamwise velocity was significantly smaller than that of oblique impingement. The overall profiles of the normalized turbulent stresses were similar regardless of impinging angles. However, the maximum stress points for α = 0° were higher from the wall than those of α = 45°. It can be explained that the vertical axis is a relative value and the jet half width for α = 0° is smaller than that for α = 45°. It can also be seen that the shear layer becomes thick after θ = 90°. Interestingly, the normalized turbulent stress profiles showed a similar trend to that of the 2D curved wall jet^12^, unlike the previous study^9^. This is because, in this study, more accurate streamwise and wall-normal turbulence fluctuation components were obtained by applying the curvilinear coordinate system. The Reynolds stress profiles show that self-similar profiles, such as the ensemble-averaged streamwise velocity, cannot be achieved in turbulent stresses. The relative turbulent stresses become discernible when the wall jet moves to the downstream direction. In particular, in the normal impingement, the maximum relative streamwise and wall-normal turbulence stresses at θ = 15° and θ = 135° differed by six-fold. This means that the maximum streamwise velocity in the downstream region for normal impingement is significantly smaller than that for oblique impingement, but the real value of the turbulent stresses for α = 45° is significantly larger, as can be seen in Fig. 10.

#### Features in the azimuthal planes

**Figure 12:**
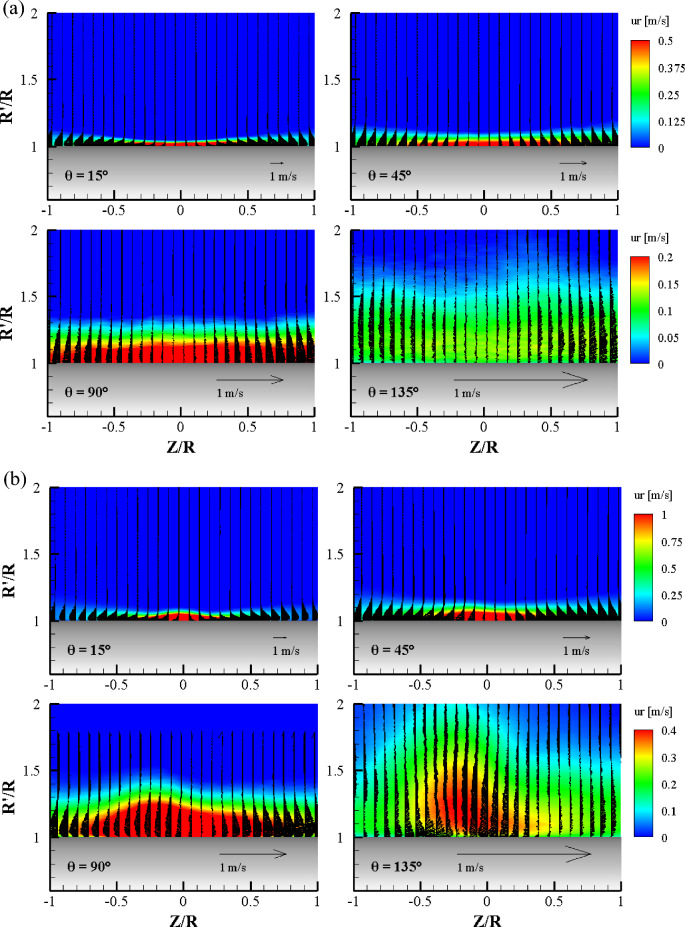
Ensemble-averaged streamwise velocity field with vectors (wall-normal and transverse velocity components) at the radial plane for each θ for (**a**) α = 0° and (**b**) α = 45°.

Figure 12 shows the ensemble-averaged 3D velocity fields obtained at various azimuthal planes. The streamwise velocity component (u_r_) is color-coded, while the wall-normal (v_r_) and transverse (w) velocity components are represented as arrow vectors. Considering that the nozzle diameter (d = 3 mm) was considerably smaller than the radius of the cylinder (R = 26 mm), the impinged jet spread quickly in all directions. Figure 12a shows the streamwise velocity fields when the jet impinges normal to the cylinder wall (α = 0°). At the θ = 15° plane, the development of the wall jet along the transverse direction was clearly visible. At the Z/R = 0 position, the thickness of the shear layer exhibited a minimum value (1 mm thickness), which is one-third the diameter of the jet nozzle. The wall jet velocity profile in Fig. 6a at θ = 15° demonstrates the high spatial resolution of the LPT data. The maximum velocity point was 0.13 mm from the cylinder surface. The transverse wall jet (depicted as vector arrows) increased linearly, like a plane wall jet, along both side directions (+ and − Z directions) from the center point. It should be noted that the magnitude of the transverse velocity was comparable to that of the streamwise velocity component. However, the maximum w-component velocity of the wall jet increased at first and then decreased because the impinged jet spreads radially like a source flow at θ = 0°. At the θ = 45° plane, the wall jet became thicker than that of the θ = 15° plane, and the velocity magnitude of the transverse wall jet decreased to half of the streamwise velocity magnitude. This means that the streamwise wall jet has a higher momentum than that of the transverse wall jet owing to the Coanda effect. At the θ = 90° plane, the 3D velocity field shows different features to that of the previous.two radial planes. The thickness of the 3D curved wall jet became 7.8 mm (7.8 times thicker than that of the θ = 15° plane) due to fluid entrainment. It is interesting that the thickness of the high-momentum zone was the same over the entire Z/R range in the figure. A high streamwise momentum zone appeared not only at the center (Z/R = 0) but also on both sides (Z/R = − 1.0 and + 1.0), and relatively weak transverse wall jet profiles were observed with a symmetric shape of development from the center plane. At the θ = 135° plane, the 3D velocity field was not entirely symmetrical with respect to the center location. It can be seen that the thickness of the wall jet exhibits a wavy nature. This can be related to the streamwise vortex structures, as already reported in the study of 2D curved wall jets^13^. However, the current results show clear flow characteristics, such as the 3D wall jet still attached to the wall and the thickness of the wall jet increasing rapidly downstream. The outward transverse wall jet still exists, although the w-component velocity is very weak (less than 0.1 m/s). In conclusion, the normally impinged jet onto a circular cylinder wall is similar to the impinging jet on a flat surface. However, owing to the Coanda effect, the streamwise momentum is stronger than the transverse momentum when the 3D curved wall jet develops on the cylinder wall. Similar profiles were clearly visible in the transverse wall jet along the direction of the cylinder axis.

Figure 12b shows the ensemble-averaged streamwise velocity fields when the jet impinges obliquely to the cylinder wall (α = 45°). It is surprising that the ensemble-averaged 3D mean velocity field of the oblique impinging jet shows a very different development of the 3D curved wall jet on the cylinder wall than that of the normal impinging jet. As can be seen in the instantaneous whole flow field (Fig. 2), most of the impinged jet moved in one direction and wrapped the cylinder completely. At the θ = 15° plane, the wall jet development along the cylinder axis (transverse direction) was clearly visible. At the Z/R = 0 position, the thickness of the shear layer is at its minimum for normal impingement; however, it is at its maximum for oblique impingement. The thickness of the high streamwise momentum zone at the center position was twice that of the normal impingement case. Note that the velocity contour range was twice that in Fig. 12a. The transverse wall jet grew linearly as a plane wall jet along both side directions from the center point. It is interesting to note that the development of the transverse wall jet along both side directions was similar to the normal impingement case in terms of thickness and velocity magnitude. At the θ = 45° plane, the wall jet flattened and the thickness of the shear layer increased by a factor of 1.5, as compared to the θ = 15° plane. It should be noted that the symmetric development of the transverse wall jet along the cylinder axis direction starts from the center plane, Z/R = 0. However, the highest thickness of the streamwise wall jet occurred at Z = − 0.1. As observed at the θ = 15° plane, the thickness of the transverse wall jet near the center was thinner than that of the streamwise wall jet. This means that the radial flow has a higher momentum than the transverse momentum owing to the cylinder curvature. At the θ = 90° plane, the 3D velocity field exhibited different features to that of the previous two radial planes and that of normal impingement. The 3D curved wall jet exhibited a distinct peak thickness in the radial plane. The peak was expected to be at the center (Z = 0), but it was located at Z/R = − 0.25. The location is more skewed than the θ = 15° plane. An irregular outward transverse wall jet was observed, but the branching location was not the center plane but was at Z/R = 0.12. There are two possible explanations for this phenomenon, namely, that the ensemble of instantaneous velocity fields is insufficient or the attached half-elliptic jet changes shape, similar to the well-known axis switching of an elliptic jet^25,26^. At the θ = 135° plane, the most surprising feature of the 3D velocity field was observed. It was evident that the spread wall jet returned to the main stream of the attached 3D curved wall jet on the cylinder. This unique kinematic supports the estimation of the axis change phenomenon of the half-elliptic jet. At Z/R = 0.25, the streamwise wall jet thickness exhibited a more pronounced peak whose thickness was almost nine times the jet nozzle diameter. It can be seen that the transverse wall jet changed direction and was joined to the maximum thickness location. This is the first observation of the flow characteristics of a curved 3D wall jet. Owing to the Coanda effect, suction pressure can appear in the high radial velocity region near the wall, and a reverse transverse wall jet could occur. The wall jet velocity profiles along the center plane were obtained at Z/R = 0, so that the thickness of the wall jet was lower than the peak at Z/R = − 0.25. During the experiments, the jet nozzle was properly located according to the defined coordinate system. Further measurements with a large number of ensembles are needed, as well as the unsteady nature of the 3D curved wall jet in the oblique impingement case.

**Figure 13:**
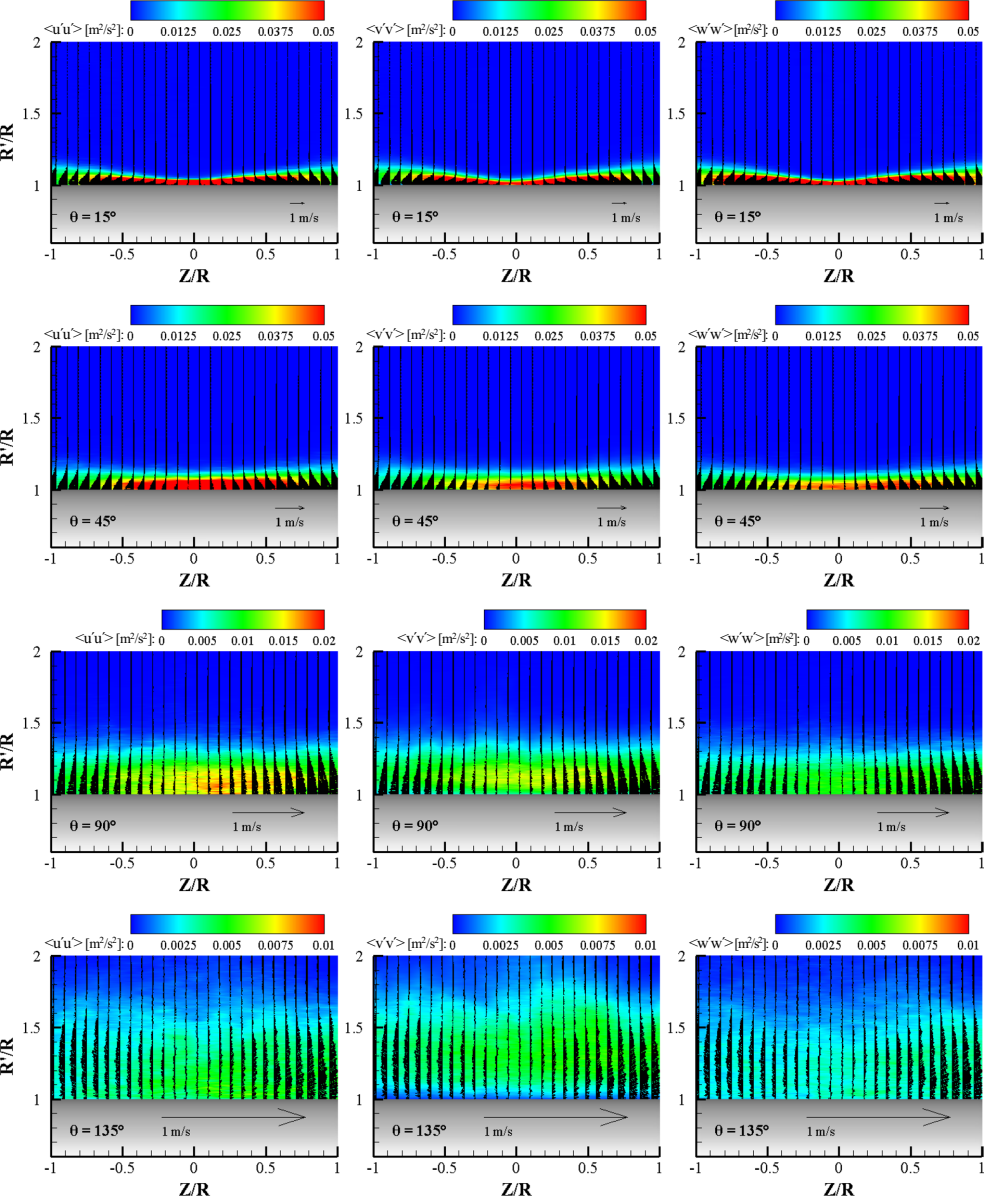
Contours of the Reynolds stress component in the same terms with vectors (wall-normal and transverse velocity components) at the radial plane for each θ for α = 0°.

Figure 13 shows the contours of the ensemble-averaged turbulent normal stress components, < u′u′ > , < v′v′ > , and < w′w′ > , at various radial planes for α = 0°. Mean velocity vector fields in the plane are added to explain the Reynolds normal stress distribution with the wall jet layer. At the θ = 15° plane, all the normal stresses showed similar contours and coincided with the shear layer profile. Additionally, all normal stress components exhibited similar magnitudes. At the maximum normal stress point (R′/R = 1.05, Z/R = 0), the magnitudes of three normal stress components are within 0.05 ± 0.005 m^2^/s^2^ which supports the local isotropy in a quantitative manner. The turbulent structure in the wall jet is inhomogeneous, however, turbulent normal stress shows an isotropic nature at local points in the wall jet. This nature supports the existence of self-preserving characteristics in the mean velocity profile, as observed in Fig. 8. At the θ = 45° plane, the normal stresses no longer have an isotropic nature. The streamwise component of normal stress, < u′u′ > , exhibited a higher value than the wall-normal and transverse components of normal stress, because the attached wall jet has a higher momentum in the streamwise direction than in the spanwise direction. The high normal stress region becomes narrow and centers to the Z/R = 0. In this region, the normal stress value is comparable to that in the θ = 15° plane. At the θ = 90° plane, the magnitude of turbulent normal stresses decreased rapidly with an increase in the wall jet thickness, showing a non-isotropic nature. The streamwise normal stress exhibited the highest value and was followed by the wall-normal component and then the transverse normal stress. At the center region of the wall jet, the shear layer was attached to the wall. A high value streamwise normal stress region appeared near the wall, however, the maximum < v′v′ > was located above the wall due to the centrifugal force exerted on the 3D curved wall jet. The low normal stress < w′w′ > value of the transverse component in this plane means the wall jet moves mostly in the streamwise direction. At the θ = 135° plane, all the normal stress values quickly decreased. In this plane, the wall jet was still attached in terms of the ensemble average and the streamwise normal stress was observed to be high near the wall. However, the magnitude of the wall-normal component of the turbulent normal stress, < v′v′ > , was comparable to that of < u′u′ > and the contour seems to be levitated from the wall. The transverse normal stress component was very weak in this plane and means that the wall jet is moving up and down in this plane.

**Figure 14:**
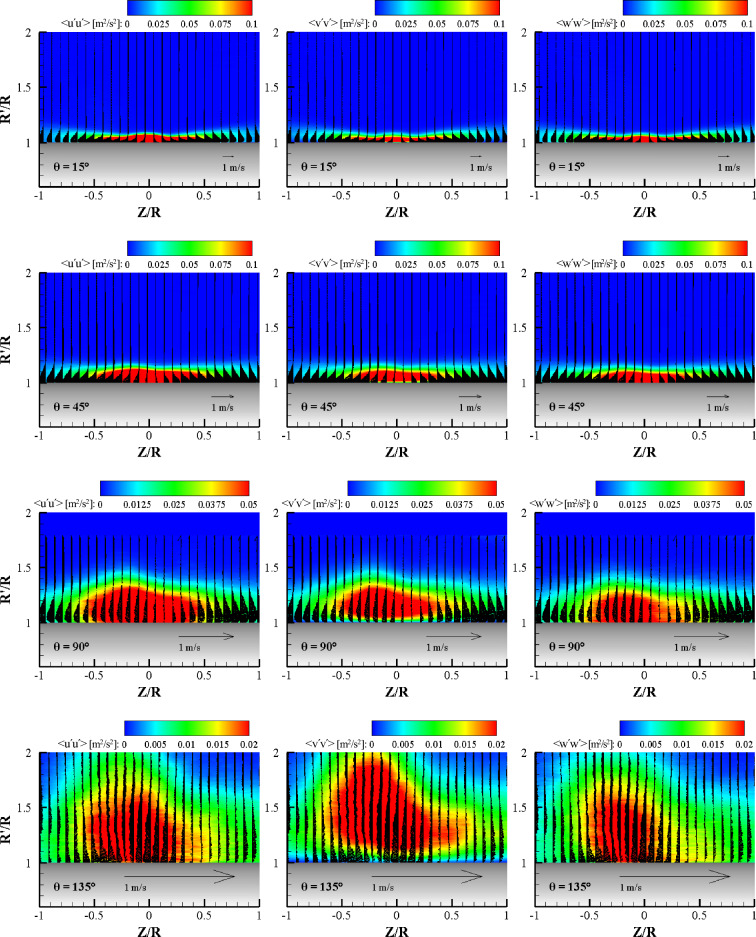
Contours of the Reynolds stress component in the same terms with vectors (wall-normal and transverse velocity components) at the radial plane for each θ for α = 45°.

Figure 14 shows the contours of the ensemble-averaged turbulent normal stress components at various azimuthal planes for α = 45°. Compared to the normal impinging case (α = 0°), the turbulent normal stress contours for oblique impinging are relatively different, especially in the far field (large cylinder angle) of the 3D curved jet. This behavior can be expected because of the unique development of a 3D curved wall jet on the cylinder wall, as shown in Fig. 12. Similar to the axis switching of an elliptic jet, the fluid flow is suddenly elongated in the wall-normal direction, and the spread wall jet in the transverse direction moves back to the center plane in the streamwise direction^25^. At the θ = 15° plane, all the normal stresses have similar contours and coincide with the shear layer profile. Furthermore, all the normal stress components exhibit similar magnitudes. Local isotropy in turbulence appears as observed in the case of normal impingement. One difference is that the normal stress contours have a peak at the center plane, similar to the mean streamwise velocity contour for α = 45°. It should be noted that the contour range is double that of the α = 0° case. The contour has a unit of m^2^/s^2^, and a high normal stress value for α = 45° is generated by a high mean velocity because most of the wall jet flows in one direction after the oblique impingement. An interesting feature can be found between the < u′u′ > and < v′v′ > distributions near the center plane. The thickness of the high < v′v′ > magnitude region is lower than that of the < u′u′ > region. This means that local isotropy is not valid in the upper portion of the wall jet at the center plane. The < v′v′ > contour appears to match well with the development of the side wall jet, so that the reduction of < v′v′ > can be explained by the transverse elongation effect. The < w′w′ > contour depicts the average of the < u′u′ > and < v′v′ > contours. At the θ = 45° plane, unlike the case of α = 0°, the normal stresses have a clear isotropic nature. The streamwise component of normal stress, < u′u′ > , exhibits a slightly larger high value area than those of the wall-normal and transverse components of normal stress, but exhibits mostly the same area and magnitude. This nature of isotropic turbulence strongly supports the existence of self-preservation in the mean velocity profiles of 3D curved wall jets. In this radial plane, the range of the normal stress contour is the same as that of the θ = 15° plane. Compared to the case of α = 0°, the high normal stress region is narrower and thicker because of the strong streamwise momentum for α = 45°. At the θ = 90° plane, with an increase in the wall jet thickness, the turbulent normal stress contours become thicker and coincide with the shear layer profile. The isotropic turbulence nature is visible in this plane, as observed in the similarity mean velocity profile in the center plane. The streamwise normal stress has a slightly larger area of high-magnitude contours than those of < v′v′ > and < w′w′ > . High-value regions of streamwise normal stress and transverse normal stress are attached to the cylinder wall; however, the high < v′v′ > value region is located above the wall owing to the centrifugal forces by the streamline curvature. Note that the range of the contour is half that of the previous radial planes. At the θ = 135° plane, the value of all normal stresses decrease to 40% of that in the previous plane. It is surprising that local isotropy still exists despite the abrupt elongation of the flow structure in the wall-normal direction. In this plane, the wall jet is firmly attached to the cylinder wall. However, the wall-normal component of the turbulent normal stress, < v′v′ > contour, is levitated from the wall owing to centrifugal force.

**Figure 15:**
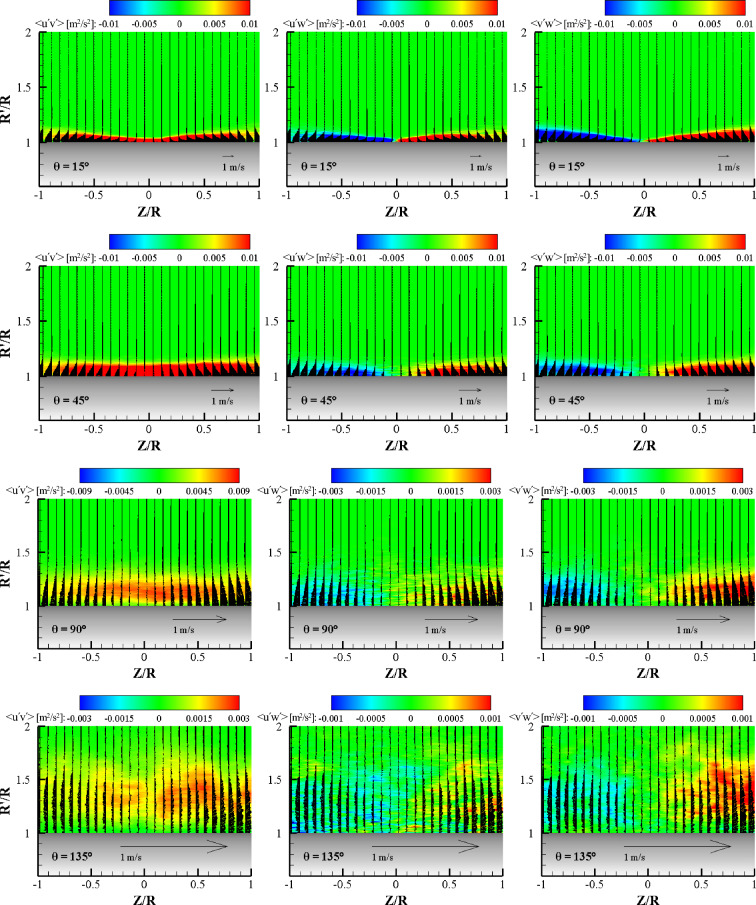
Contours of the Reynolds stress component in mixed terms with vectors (wall-normal and transverse velocity components) at the radial plane for each θ for α = 0°.

Figure 15 shows contours of the ensemble-averaged turbulent shear stress components, < u′v′ > , < u′w′ > , and < v′w′ > , at various azimuthal planes for α = 0°. Mean velocity vectors are represented to describe the Reynolds shear stress distribution with the wall jet layer. At the θ = 15° plane, all the turbulent shear stresses exhibit similar absolute magnitudes. The contours coincide with the shear layer profile. The < u′v′ > shear stress is mostly positive, except for very near the wall. At this plane, the mean streamwise velocity gradient, ∂u_r_/∂y, is mostly negative, meaning that turbulence can be generated in the entire wall jet layer. The < u′w′ > and < v′w′ > contours exhibit positive and negative values according to the Z-direction sign. The < v′w′ > shear stress contour coincides exactly with the growth of the transverse wall jet development. In the positive Z-direction, positive < v′w′ > with negative ∂w/∂y generates turbulence. At the θ = 45° plane, the Reynolds shear stress component < u′v′ > contour exhibits a positive value in the entire wall jet area and the thickness of the contour is flat and higher than that of the θ = 15° plane. Note that the contour range is the same for both planes. Both < u′w′ > and < v′w′ > have comparable positive and negative values with the < u′v′ > shear stress, however, there are no < u′w′ > and < v′w′ > shear stresses near the center plane because of the strong streamwise flow momentum in this region. At the θ = 90° plane, the contours of all the turbulent shear stresses are extended to the wall-normal direction with an increase of the wall jet thickness. It should be noted that the contour range of < u′v′ > is three times larger than those of < u′w′ > and < v′w′ > . This nature can be easily understood since the flow in the streamwise direction is dominant. At the center region of the wall jet, < u′v′ > exhibits a high value but the maximum point is moved to the normal direction because of centrifugal force. Again, no < u′w′ > and < v′w′ > shear stresses were visible near the center plane due to very low velocity in the transverse direction. At the θ = 135° plane, all the Reynolds shear stress values decreased quickly. In this plane, the < u′v′ > shear stress was highest, followed by < v′w′ > and < u′w′ > . This means that the turbulence structure is highly inhomogeneous.

**Figure 16:**
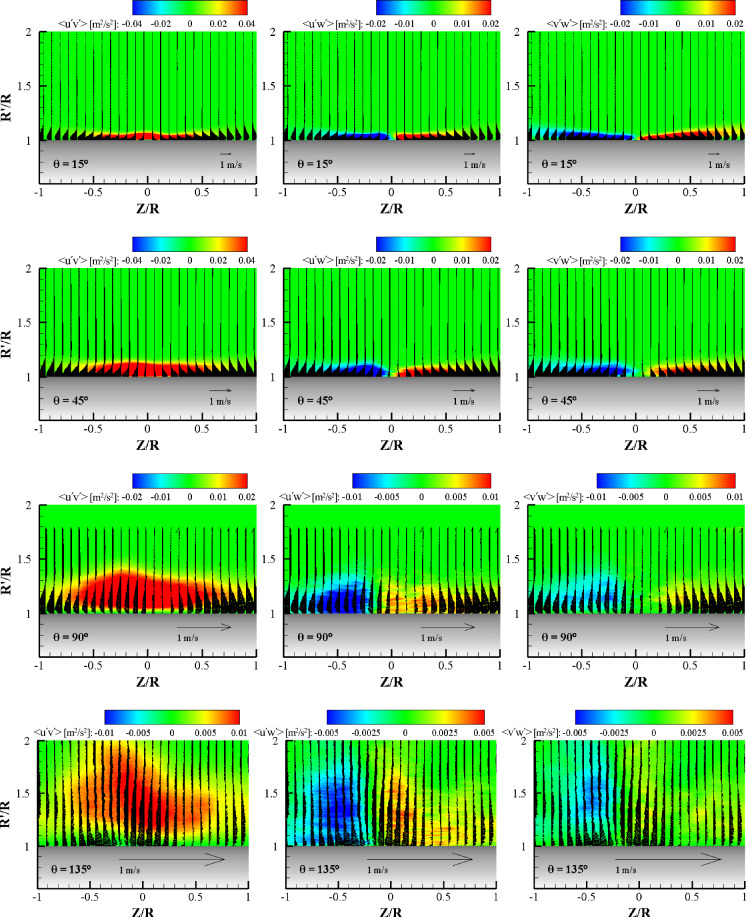
Contours of the Reynolds stress component in mixed terms with vectors (wall-normal and transverse velocity components) at the radial plane for each θ for α = 45°.

Figure 16 shows the contours of the ensemble-averaged turbulent shear stress components for α = 45°. The overall characteristics of the Reynolds shear stress components were similar to those observed in the normal impingement case. At the θ = 15° plane, < u′v′ > exhibits a value two times higher than that of the other shear stress components. At the center plane (Z = 0), the < u′v′ > shear stress exhibited the highest value and a peak similar to the wall jet profile. The < u′w′ > value at Z = 0 is zero, but the largest thickness near the center plane is due to the dominant streamwise flow. However, the < v′w′ > contour thickness increases with the transverse wall jet profile. At the θ = 45° plane, the Reynolds shear stresses show a similar nature to the normal impingement case. The < u′v′ > shear stress component was almost twice that of the < u′w′ > and < v′w′ > shear stress values. This can be explained by the fact that.the attached wall jet has a higher momentum in the streamwise direction than in the spanwise direction. Note that the high < u′v′ > shear stress region becomes narrow, and the two other components of shear stress, < u′w′ > and < v′w′ > , have zero values at Z/R = 0. In this region, the magnitude of the shear stress is comparable to that in the θ = 15° plane. At the θ = 90° plane, with an abrupt increase in the wall jet thickness in the negative Z location, the peak values of < u′v′ > and the zero value region of < u′w′ > are shifted to the negative Z location. However, the zero value of the < v′w′ > region is still at the Z = 0 position because this shear stress is related to the transverse wall jet. At the θ = 135° plane, the contours of the Reynolds shear stresses are dramatically changed with a sudden increase in the wall jet thickness along the wall-normal direction. In this plane, the peak of the < u′v′ > contour and the zero value regions of < u′w′ > and < v′w′ > are the same at Z/R = − 0.2. As previously mentioned, at the exact region where this location is shifted to the negative Z direction, the turbulent shear stress contours are well matched with the mean velocity contours. Compared to normal impingement, the turbulent structure of oblique impingement is relatively different in shape and magnitude.

### 3D vortex structures on the cylinder surface

**Figure 17:**
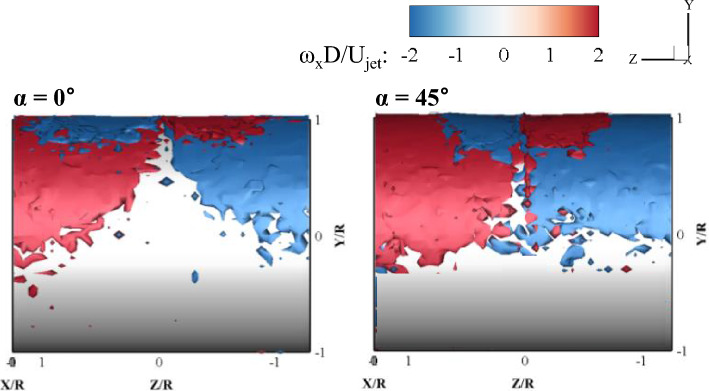
Ensemble-averaged streamwise vortex structures near the cylinder wall.

**Figure 18:**
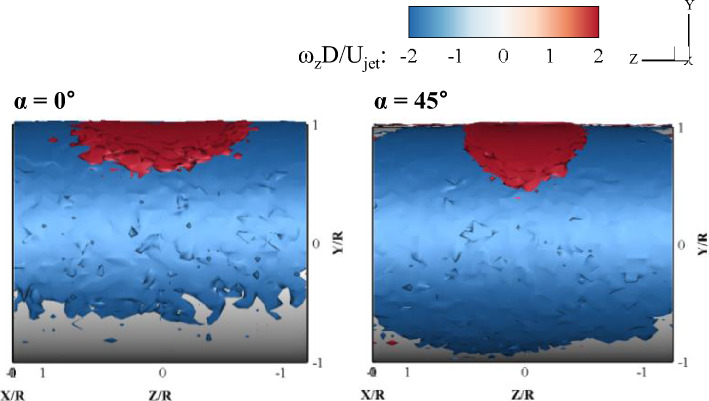
Ensemble-averaged spanwise vortex structures near the cylinder wall.

Figures 17, 18 show the ensemble-averaged 3D streamwise (ω_x_) and spanwise (ω_z_) vorticity structures near the cylinder surface (1.0 < R′/R < 1.05), respectively, in the front view obtained with the radial grid. In the radial grid, the velocity gradients in both vortices had small derivative errors because the flow fits the coordinates. A streamwise vortex is a flow structure in which the flow rotates counterclockwise (positive value) and clockwise (negative value) on both sides around the X axis (Z = 0), and is obtained by the equation:

2 $${\omega }_{x}=\frac{\partial w}{\partial y}-\frac{\partial {v}_{r}}{\partial z}$$

where *y* and *v*_*r*_ represent the wall-normal direction and velocity in the curvilinear coordinate system, respectively, and *z* and *w* indicate the spanwise direction and velocity in the curvilinear coordinate system, respectively. For both impinging angles, the streamwise vortex structures are related to the spreading flow in the transverse direction. Positive vorticity values appeared near the cylinder wall when lateral spreading on the left-hand side of the centerline occurred. On the right-hand side of the centerline, both the Z-axis and the transverse velocity had negative values, so that the streamwise vortex has a negative value. Along the streamwise wall jet flow, the ensemble-averaged streamwise vorticity was nearly zero because the attached curved jets no longer spread laterally and were completely randomly generated even in the presence of a streamwise vortex structure in the instantaneous flow field. The ensemble-averaged vorticity is a value obtained at each point and is a gathering of vortex structures with very small velocity gradients. It is interesting to note that opposite-sign vortex structures were observed in the upper layer of the wall jet just after impingement. This means that a counter-rotating streamwise vortex pair appears near the impingement point. When the impinging angle was 45°, the gap between the vortex pair structures was closer than that of the impinging angle of 0°, as indicated in Fig. 17. This means that when the impinging angle is 45°, the lateral momentum flux is relatively low, as compared to the streamwise momentum flux.

Figure 18 shows the 3D ensemble-averaged spanwise vortex structure in the jet impinging onto the circular cylinder, and is obtained by the equation:

3 $${\omega }_{z}=\frac{\partial {v}_{r}}{\partial x}-\frac{\partial {u}_{r}}{\partial y}$$

where *x* and *u*_*r*_ denote the streamwise direction and velocity in the curvilinear coordinate system, respectively. Because ∂u_r_/∂y is the dominant term in the spanwise vorticity, negative spanwise vortices widely appeared on the cylinder wall. The negative spanwise vorticity near the wall represents the boundary layer, while the positive spanwise vorticity can appear to be higher than the maximum velocity point, as shown in Fig. 6. Interestingly, the shape of the positive spanwise vortex structure appears differently depending on the impinging angle. A semicircle positive vortex structure at α = 0° and a V-shaped positive vortex structure at α = 45° were formed on the cylinder surface. In the case of α = 0°, the jet flow was initially widely spread in the spanwise direction, and then the length in the spanwise direction gradually decreased and moved toward the streamwise direction; thus, a semicircular jet flow was observed. In contrast, for α = 45°, because the jet impinged with an inclination angle, the jet flow moved in a long and thick layer toward the streamline, and a V-shaped flow was observed.

We showed instantaneous vorticity field with iso-surface of the Q-criterion in Fig. 2. Instantaneous vorticity field is good to find coherent structures. However, this paper is focused on the ensemble averaged statistical quantities in the 3D turbulent curved wall jet flow. The ensemble-averaged vorticity field near the surface reveals a strong shear near the cylinder surface and supports the similarity (Reynolds analogy) between momentum and heat transfer characteristics which we already published^1^.

## Conclusions

The ensemble-averaged velocity and turbulence characteristics of a 3D curved wall jet on a circular cylinder were experimentally studied. Time-resolved volumetric LPT was used to measure the 3D and three-component flow fields. Using advanced LPT technology VT-STB, a dense 3D flow field of the impinging jet flow with a high dynamic velocity range was obtained, making it possible to accurately track both the slow surrounding flow and the relatively fast particles within the jet. Cartesian and radial grids were used for bin averaging to obtain the mean flow field and respective Reynolds stresses. The difference between the vector fields and profiles of the ensemble-averaged velocity, according to the bin-averaging method, was studied. Although Cartesian binning had a higher number of nodes, the velocity vectors and profiles near the curved surface were clearer with radial binning. In particular, the difference was evident in the θ > 90° region where the wall jet slowed down, and the direction of the u velocity was reversed. On the other hand, radial binning could not resolve the entrained flow features around the curved wall jet, but was suitable for studies requiring velocity profiles and distributions on curved surfaces of the wall jet region.

The ensemble-averaged velocity and turbulent stress fields in the center plane of the wall jet were obtained. Both impinging angles showed a clear Coanda effect through the attachment of the flow up to the maximum downstream region of each measurement volume. In particular, when α = 45°, the flow separation point could not be found in the ensemble-averaged velocity field even at a cylinder angle of 180°. When the mean streamwise velocity profiles were scaled by the maximum streamwise velocity and the jet half-width, both impinging angles showed similar mean velocity profiles, clearly showing that the self-preserving phenomenon existed. This characteristic was first discovered in this study, proving that the 3D curved wall jet can be a canonical flow of wall turbulence. The rate of change of the flow direction of the maximum velocity and the jet half-width was in good agreement with the exponential functions and was dependent on the impinging angle.

The 3D ensemble-averaged velocity field and turbulent stress tensor components in various azimuthal planes were analyzed for the two impinging angles at a fixed Re. The flow and turbulence characteristics of the jet impinging on the cylinder were significantly different with respect to the impinging angle, especially in the downstream region. A surprising result is that the attached wall jet, having a half-elliptic shape, was abruptly thickened in the wall-normal direction, similar to the axis change phenomenon in turbulent elliptic jets in the case of oblique impingement. It was clearly observed that the spread wall jet returned to the main stream of the attached 3D curved wall jet on the cylinder. This unique kinematic supports the conjecture of the axis change phenomenon of the half-elliptic jet. In the jet-impinging region, the flow spreads in all directions with high mean vorticity values. With the development of a 3D curved wall jet, both the Coanda effect and centrifugal force characterize the flow behavior. The streamwise and spanwise vorticity contours obtained from the average velocity gradient data shows that there is a significant difference between the flow characteristics immediately after the jet impinges, and the flow characteristics formed as the flow moved along the wall. The turbulent structure in the wall jet is inhomogeneous; however, turbulent normal stress exhibits an isotropic nature at local points in the wall jet. This supports the existence of self-preserving characteristics in the mean velocity profile. The volumetric ensemble-averaged Reynolds stress tensor revealed strong inhomogeneous turbulence in the 3D curved wall jet. The overall characteristics of the Reynolds shear stress components were similar for both impinging angles in the range of early development of the wall jet, 0 < θ < 90°. In the early stages of the 3D curved wall jet, the normalized turbulent normal and shear stress profiles in the center plane were similar to those of a 2D curved wall jet. However, in the downstream region where θ is larger than 90°, the turbulent stress tensor profiles were significantly different with impinging angles. The present experimental data provide many new findings on fluid dynamics when a round jet impinges on a cylinder wall and can serve as benchmark data for the validation of computational fluid dynamics simulations.

## Supplementary Information

Supplementary Video 1.

## Data Availability

The datasets generated and/or analyzed during the current study are not publicly available due to the local data safety agreement but are available from the corresponding author on reasonable request.
